# *Wolbachia* strain *w*AlbB blocks replication of flaviviruses and alphaviruses in mosquito cell culture

**DOI:** 10.1186/s13071-020-3936-3

**Published:** 2020-02-10

**Authors:** O’mezie Ekwudu, Gregor J. Devine, John G. Aaskov, Francesca D. Frentiu

**Affiliations:** 1 School of Biomedical Sciences and Institute of Health and Biomedical Innovation, Queensland University of Technology, Herston, QLD 4006 Australia; 2 Department of Microbiology, Chukwuemeka Odumegwu Ojukwu University, Uli, Nigeria; 3 Mosquito Control Laboratory, Queensland Institute of Medical Research Berghofer, Herston, QLD 4006 Australia

**Keywords:** Arbovirus, Mosquito, Dengue, Zika, Ross River Virus, West Nile, Sindbis

## Abstract

**Background:**

*Wolbachia pipientis* are bacterial endosymbionts of arthropods currently being implemented as biocontrol agents to reduce the global burden of arboviral diseases. Some strains of *Wolbachia*, when introduced into *Aedes aegypti* mosquitoes, reduce or block the replication of RNA viruses pathogenic to humans. The *w*AlbB strain of *Wolbachia* was originally isolated from *Aedes albopictus*, and when transinfected into *Ae. aegypti*, persists in mosquitoes under high temperature conditions longer than other strains. The utility of *w*AlbB to block a broad spectrum of RNA viruses has received limited attention. Here we test the ability of *w*AlbB to reduce or block the replication of a range of *Flavivirus* and *Alphavirus* species in cell culture.

**Methods:**

The C6/36 mosquito cell line was stably infected with the *w*AlbB strain using the shell-vial technique. The replication of dengue, West Nile and three strains of Zika (genus *Flavivirus*), and Ross River, Barmah Forest and Sindbis (genus *Alphavirus*) viruses was compared in *w*AlbB-infected cells with *Wolbachia*-free controls. Infectious virus titres were determined using either immunofocus or plaque assays. A general linear model was used to test for significant differences in replication between flaviviruses and alphaviruses.

**Results:**

Titres of all viruses were significantly reduced in cell cultures infected with *w*AlbB versus *Wolbachia*-free controls. The magnitude of reduction in virus yields varied among virus species and, within species, also among the strains utilized.

**Conclusion:**

Our results suggest that *w*AlbB infection of arthropods could be used to reduce transmission of a wide range of pathogenic RNA viruses.
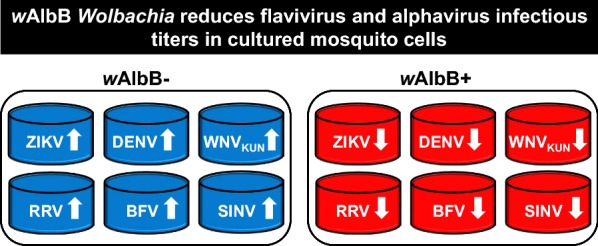

## Background

Mosquito-borne viruses contribute significantly to the global burden of infectious diseases. Two genera of viruses responsible for significant numbers of human disease cases are *Flavivirus* and *Alphavirus*. Dengue viruses (DENV) are the most important human pathogens among the flaviviruses (family *Flaviviridae*), causing an estimated 390 million infections annually among the more than 2.5 billion people at risk of infection [1, 2]. Zika virus (ZIKV) causes a mild febrile illness in adults and may result in foetal loss during pregnancy and congenital neural malformations in babies [3, 4]. West Nile virus (WNV) can cause encephalitis and is now endemic in Europe and North America [5, 6]. The Australian strain of WNV, Kunjin virus (WNV_KUN_), also can cause encephalitis [7]. Within the genus *Alphavirus* (family *Togaviridae*), Ross River virus (RRV) and Barmah Forest virus (BFV) are two of the most common infections occurring in Australia and cause arthralgia and myalgia [8]. RRV also has caused outbreaks of disease in the Pacific, resulting in tens of thousands of cases [9]. Sindbis virus (SINV) infections are associated with a rash and mild fever in humans and have caused disease outbreaks in northern Europe [10, 11].

Transinfection of mosquito vector populations with *Wolbachia* has been proposed as an arbovirus biocontrol measure that may be self-sustaining and environmentally friendly [12]. *Wolbachia* are obligate intracellular bacteria that have evolved diverse ways to manipulate reproduction in their arthropod hosts in order to invade host populations [13, 14]. It is estimated that between 40–60% of all insect species are infected with diverse strains of *Wolbachia* [15, 16]. When transinfected into *Aedes aegypti* mosquitoes, some *Wolbachia* strains block the replication and transmission of viruses such as dengue, Zika and chikungunya (CHIKV) [17–22]. The pathogen-blocking ability of *Wolbachia* has resulted in this biocontrol agent being trialled in the field in at least 12 countries (http://www.worldmosquitoprogram.org), with the aim of making native mosquito populations refractory to arbovirus transmission [22–24].

The ability of *Wolbachia* to block pathogen replication depends, in part, on the strain of bacteria being used [25, 26]. Stable infections have been established in *Ae. aegypti* with several strains, including *w*MelPop [27] and *w*Mel [18], both of which are native to *Drosophila melanogaster*. *w*MelPop over-replicates in its hosts and is highly effective in restricting replication and transmission of a broad range of human arboviruses, including DENV [17, 20], CHIKV [17, 28], yellow fever virus [17, 28] and WNV [19]. However, *w*MelPop is unlikely to invade and persist in wild populations due to its reduction of host fitness [26, 29–31]. *w*Mel blocks the replication of DENV [18, 22, 32], ZIKV [33, 34] and CHIKV [35], without significantly reducing mosquito fitness [18]. It is also able to invade and persist in mosquito populations [23, 24, 36]. However, *w*Mel can be lost from the mosquito host when exposed to heat stress [37, 38], potentially reducing the extent of virus blocking and slowing the spread of *Wolbachia* through a vector population.

The *Wolbachia* strain *w*AlbB, isolated from *Ae. albopictus* mosquitoes, has been found to be more stable than *w*MelPop and *w*Mel under high heat conditions both in the laboratory [38] and the field [39]. At high temperatures, *w*AlbB transinfected into *Ae. aegypti* mosquitoes, exhibited a high and stable density of bacteria, and high maternal transmission fidelity [38–40]. *w*AlbB has invaded caged populations of *Ae. aegypti* [41], blocks DENV transmission in at least 40% of mosquitoes [20, 40] and is currently being tested in the field in Malaysia [42].

Preliminary results from releases in Malaysia suggest that *w*AlbB can persist in field mosquitoes, be maintained at high frequencies, and may significantly reduce dengue incidence [43]. Despite *w*AlbB holding significant promise as a biocontrol agent, its ability to block the replication of a broad range of human arboviruses has not been systematically tested. Here, we test the ability of *w*AlbB to block the replication of several flaviviruses and alphaviruses in mosquito cell lines.

## Methods

### Mosquito cells and infection with *w*AlbB

The *Ae. albopictus* cell line C6/36 [44] was maintained at 28 °C in RPMI-1640 medium containing 25 Mm HEPES (Sigma-Aldrich, Castle Hill, Australia), supplemented with 10% v/v heat-inactivated foetal bovine serum (FBS, Gibco, Mt. Waverely, Australia) and 1% v/v l-glutamine (Invitrogen, Carlsbad, USA). The *w*AlbB-infected cell line, designated C6/36.*w*AlbB, was generated by introducing *w*AlbB from Aa23 *Ae. albopictus* cells [45] into C6/36 cells using the shell vial technique, according to previously published methods [46, 47]. C6/36.*w*AlbB cells were maintained in 2:1 mixture of RPMI-1640 media buffered with HEPES (Sigma-Aldrich) and Schneider’s *Drosophila* Modified medium (Lonza, Basel, Switzerland), supplemented with 10% v/v FBS and 1% v/v l-glutamine. Preliminary experiments (data not shown) indicated Schneider’s *Drosophila* Modified medium (Lonza, Basel, Switzerland) was necessary for maintenance of *w*AlbB in cell culture. All insect cells were maintained at 28 °C and subcultured in maintenance media at a 1:3 ratio once each week for C6/36.*w*AlbB cells and 1:5 ratio twice a week for C6/36 controls.

### Fluorescent *in situ* hybridization (FISH) for *w*AlbB detection

C6/36.*w*AlbB cells and C6/36 control cells without *Wolbachia* were seeded into duplicate wells in chambered slides (Bio-Basic, Ontario, Canada) and incubated for 24 h at 28 °C. Cell monolayers were washed with sterile phosphate buffered saline (PBS), fixed with ice-cold 4% paraformaldehyde (PFA) (VWR Alfa, BioStrategy, Tingalpa, Australia) at 4 °C for 30 min and then washed three times in 0.1 M phosphate buffer. The cells were dehydrated by sequential immersion of the slides, at 2 min intervals, in 70%, 95% and 100% v/v ethanol/water at room temperature. Hybridization was conducted overnight at 37 °C in a humidified container with hybridization cocktail II + 50% formamide (BioBasic, Ontario, Canada) containing 100 ng/µl of Cy5 labelled, *Wolbachia*-specific *16S* rRNA W2 oligonucleotide probe (5ʹ-CY5-CTT CTG TGA GTA CCG TCA TTA TC-3ʹ) [48], synthesized at IDT DNA (Singapore). After hybridization, the slides were rinsed in 1× SSC buffer containing 10mM dithiothreitol (DTT) (AppliChem GmbH Germany), and then twice in 0.5× SSC buffer containing 10 mM DTT. All washes were performed at 55 °C for 15 min each. Cells were then stained with 0.5 µg/ml DAPI (Sigma-Aldrich, Castle Hill, Australia) and images captured on a Zeiss epifluorescent microscope at 100× magnification. Signals from five separate microscope fields from 3 independent cell culture samples were analysed.

### Virus species and strains

WNV_KUN_ (MRM 16 strain), RRV (T48), BFV (16313) and SINV (MRM39) were obtained from the World Health Organisation Collaborating Centre for Arbovirus Reference and Research at Queensland University of Technology, Australia. We used DENV serotype 2 strain ET300 (GenBank: EF440433) as a representative strain of dengue. The following strains of Zika virus were used: a Brazilian isolate (GenBank: KU365780), the French Polynesian isolate H/PF/2013 (GenBank: KJ776791) and the African genotype reference strain MR766. All virus stocks were propagated in C6/36 cells maintained as described above but with FBS supplementation reduced to 2%. Culture supernatant was harvested 2 days following infection of cells with SINV, 3 days after RRV and BFV infections, and 4 days after WNV_KUN_ infections. Supernatants were harvested 4 days post-infection (dpi) for ZIKV strain KU365780 and 5 dpi for ZIKV strains MR766 and H/PF/2013, and DENV-2 ET300. Cell debris was removed from culture supernatants by centrifugation at 4000×*g* for 10 min at 4 °C and virus concentrated by ultrafiltration through a 100 kDa filter in an Amicon filter device (Merck Milipore, Massachusetts, USA) according to the manufacturer’s instructions. The concentrate was aliquoted into sterile 2 ml cryovials before freezing at – 80 °C.

### Virus infection experiments

C6/36 and C6/36.*w*AlbB cells were seeded into 24-well plates at 2.5 × 10^5^ cells per well and allowed to attach for 24 h at 28 °C. Infection with each virus strain was performed in triplicate wells, at multiplicities of infection (MOI) of 0.1, 1 or 10 in FBS-free RPMI-1640 medium (Sigma-Aldrich, Castle Hill, Australia). The virus was allowed to adsorb for 2 h before the inoculum was removed, the monolayers were washed twice with sterile PBS and then incubated at 28 °C in fresh maintenance media [RPMI-1640 containing 25 mM HEPES (Sigma-Aldrich) supplemented with 2% FBS (Gibco) and 1% Glutamax (Sigma-Aldrich)]. Supernatants were harvested from three independent replicate wells every 24 h for 8 days from cultures infected with flaviviruses. Because alphaviruses replicate much faster than flaviviruses, supernatants for these viruses were sampled every 8 h up to 48 h post-infection (8, 16, 24, 32, 40 and 48 h), then every 24 h until day 6 (72, 96, 120 and 144 h) and finally at day 8 (192 h).

### Plaque and immunofocus assays to determine virus titres

Infectious virus titres were determined using either plaque or immunofocus assays on Vero (African green monkey kidney) cells maintained in Dulbecco’s modified Eagle’s medium (DMEM) (Sigma-Aldrich) containing 5% v/v foetal bovine serum (FBS, Gibco) and 1% l-glutamine (Invitrogen, Carlsbad, USA) at 37 °C in an atmosphere of 5% v/v CO_2_/air. Cells were seeded in 24-well plates at 2.0 × 10^5^ cells per well and incubated overnight at 37 °C. Confluent monolayers were infected with 200 µl of serial ten-fold dilutions of virus for 2 h at 37 °C, with gentle rocking every 15 min. A 1 ml overlay (1:1 v/v) consisting of 8% w/v carboxy-methyl cellulose (CMC, Sigma-Aldrich) and Medium 199 (Sigma-Aldrich) was added to each well and plates incubated at 37 °C in an atmosphere of 5% v/v CO_2_/air. After the desired length of incubation (i.e. 2 days for SINV, 3 days for RRV and BFV, 4 days for KUNV and ZIKV KU365780, and 5 days for ZIKV MR766 and P13F/251013-18), overlay media was removed and cell monolayers were washed twice in PBS. Cells then were stained with 300 µl of 0.05% w/v Crystal violet in 1% v/v formaldehyde and PBS for 1 h, rinsed with water, dried and plaques counted.

As DENV did not produce plaques reliably with the protocol above, infectious titres were determined using immunofocus assay. Initial steps were performed as above before proceeding with the following modifications. Five days post infection, the CMC overlay was removed, and cell monolayers fixed with ice-cold (1:1 v/v) acetone-methanol (Thermo Fisher Scientific, Brisbane, Australia). Blocking was performed by the addition of 200 µl of 5% w/v skim milk powder in PBS for 1 h at 37 °C. DENV-infected cells were detected using the anti-*Flavivirus* monoclonal antibody 4G2 (TropBio, Cairns, Australia) as the primary antibody, followed by horseradish peroxidase (HRP)-conjugated goat anti-mouse IgG (Invitrogen, Carlsbad, USA) as a secondary. Infectious foci were detected using SigmaFast with DAB (Sigma-Aldrich), after the manufacturer’s instructions. Plaque and immunofocus assays were performed in duplicate for each sample.

### Analyses

Virus titres were log_10_-transformed and general linear models were used to test for statistically significant differences. The Chi-square test of association, Fisher’s exact test, and a general linear model were used to compare the results from cell lines separately for each time point and for each MOI. Statistical analyses were performed using the IBM SPSS Statistics software (version 23.0) (SPSS Inc., Chicago, USA) and GraphPad Prism Version 7.00 (GraphPad Software, La Jolla, California USA, 2008). To enable graphing of virus titre values of 0 (no plaques), 1 was added to all values and the resulting number log_10_-transformed.

## Results

### Stable infection of C6/36 cells with *Wolbachia* strain *w*AlbB

**Fig. 1:**
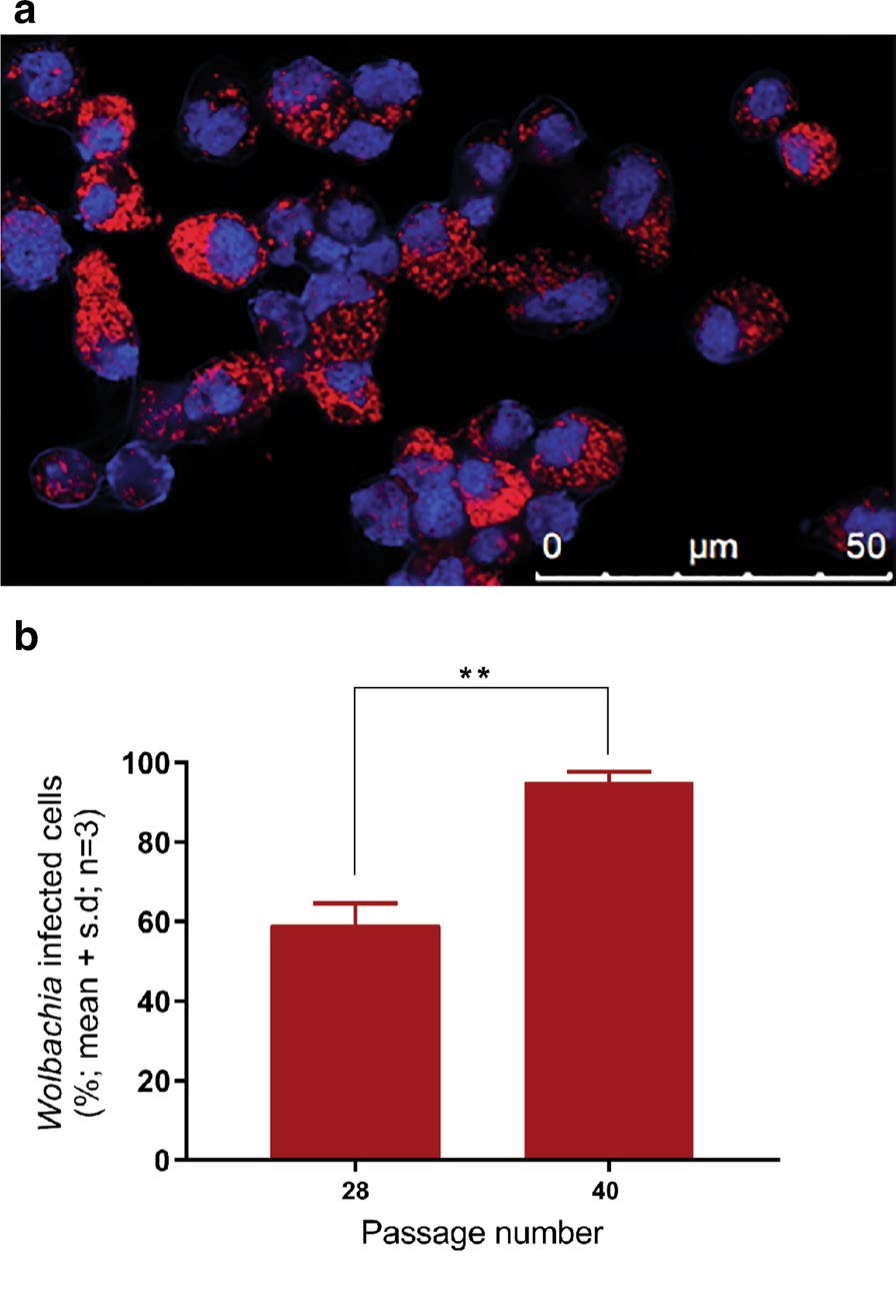
Detection of *Wolbachia w*AlbB by Fluorescent in situ hybridization of C6/36.*w*AlbB cells. **a** Carbocyanine5-labelled oligonucleotide probe corresponding to nucleotide sequences in *Wolbachia 16S* rRNA within the cytoplasm of the host cell (red). Cell nuclei stain blue with DAPI. **b** Proportion of cells containing *Wolbachia w*AlbB detectable by FISH between passages 28 and 40. Images were taken at a magnification of 100×. Error bars represent the standard deviation of the mean of three independent cell culture samples. Statistical significance was calculated by Mann Whitney test (*P* < 0.05, denoted by **)

The presence of *Wolbachia* in the cytoplasm of C6/36.*w*AlbB cells was confirmed using FISH (Fig. 1a). The density of *w*AlbB in the cytoplasm of infected C6/36 cells was less than 40% in early cell passages (P 1-20; data not shown), as found by other groups [49]. However, by passage 40, the percentage of cells containing *w*AlbB had increased from approximately 60% in passage 28 to more than 95% (*P* < 0.01 by Mann Whitney test; Fig. 1b).

### *Wolbachia* strain *w*AlbB blocks *Flavivirus* replication *in vitro*

**Fig. 2:**
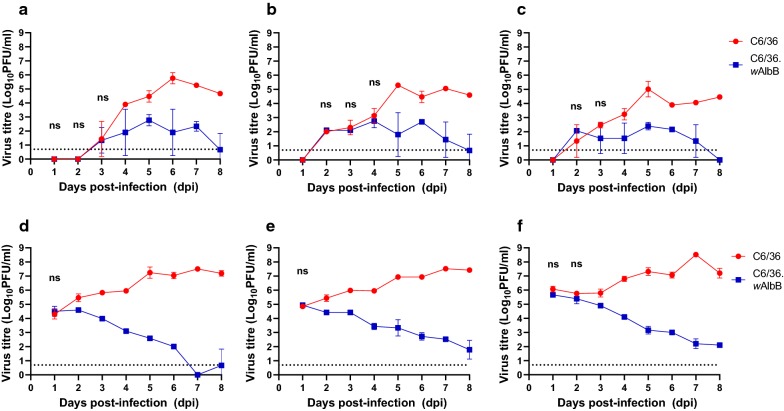
Kinetics of virus production following infection of C6/36 and C6/36-*w*AlbB cells with DENV 2 strain ET300 and WNV_KUN_ at MOI of 0.1 (**a**, **d**), 1 (**b**, **c**) and 10 (**c**, **f**). Means and standard deviations (error bars) for each time point are shown (*n* = 3 wells per time point). *Abbreviations*: PFU, plaque forming unit; ns, virus yields that are not significantly different (*P* > 0.05) between cell lines. Limit of detection for the plaque assay is shown as a dotted line

**Fig. 3:**
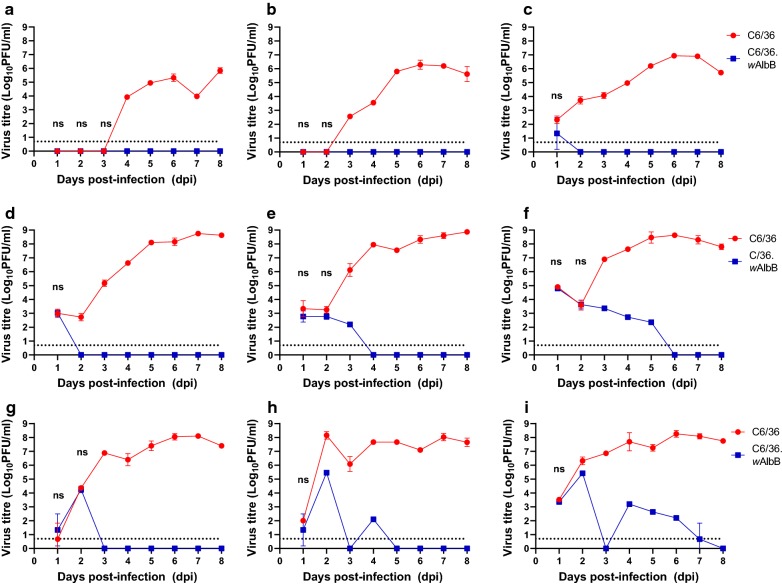
Kinetics of virus production following infection of C6/36 and C6/36.*w*AlbB cells with three strains of ZIKV at MOI of 0.1, 1 and 10 (left to right). African strain MR766 (**a**–**c**), Brazilian strain KU365780 (**d**–**f**), and French Polynesian strain H/PF/2013 (**g**–**i**). Means and standard deviations (error bars) for each time-point are shown (*n* = 3 wells per time-point). *Abbreviations*: PFU, plaque forming unit; ns, virus yields that are not significantly different (*P* > 0.05) between cell lines. Limit of detection for the plaque assay is shown as a dotted line

All flaviviruses tested replicated to lower titres in C6/36.*w*AlbB cells compared to *Wolbachia*-free C6/36 controls, regardless of MOI. Although titres from *Wolbachia*-infected and control cells were similar at early time points (1–3 dpi, Fig. 2), titres of DENV produced in C6/36.*w*AlbB were reduced by an average of 2–3 logs by 8 days post-infection (dpi) (Fig. 2a–c). Titres of WNV_KUN_ were reduced by almost 5 logs, particularly at later time points during infection (6–8 dpi) (Fig. 2d–f), although virus remained detectable until the end of the experiment. Only with ZIKV did we observe a complete cessation in replication due to *w*AlbB presence (Fig. 3). Replication of ZIKV African strain MR766 was reduced to a point where no infectious virus particles could be detected by plaque assay, except for 1 dpi post-infection and at the high MOI of 10 (Fig. 3a–c). Titres of Brazilian strain ZIKV-KU365780 were reduced by at least 6 logs at 8 dpi across all MOI (Fig. 3d–f). For the French Polynesian strain H/PF/2013, initial replication in C6/36.*w*AlbB cells resulted in virus titres comparable to titres from control C6/36 cells, but titres became undetectable at 3 dpi (Fig. 3g–i). For both Brazilian and French Polynesian ZIKV strains, we observed that the higher the MOI the longer it took before infectious virus disappeared from C6/36.*w*AlbB cells.

### *Wolbachia* strain *w*AlbB blocks *Alphavirus* replication *in vitro*

**Fig. 4:**
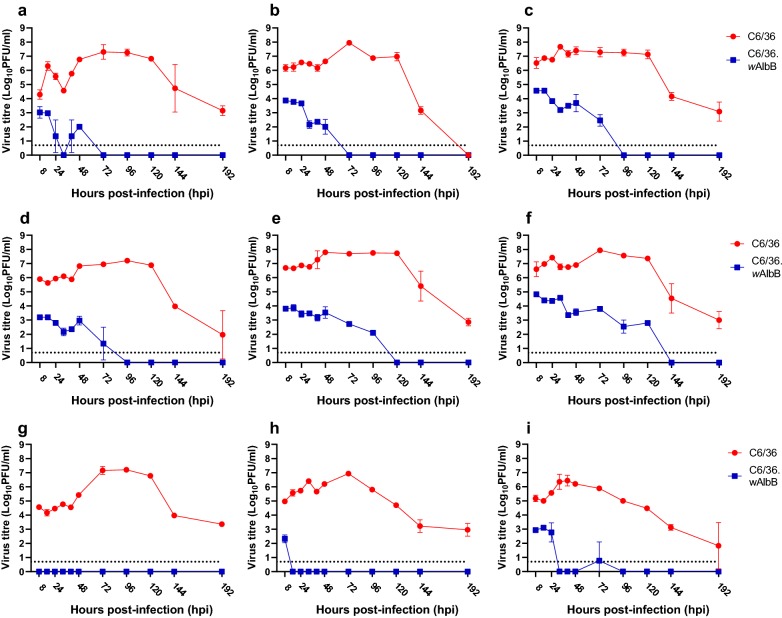
Kinetics of virus production following infection of C6/36 and C6/36.*w*AlbB cells with the alphaviruses BFV (**a**–**c**), SINV (**d**–**f**) and RRV (**g**–**i**) in C6/36 at MOI of 0.1, 1 and 10. Means and standard deviations (error bars) for each time-point are shown (*n* = 3 wells per time-point). *Abbreviations*: PFU, plaque forming unit. Limit of detection for the plaque assay is shown as a dotted line

The replication of the three alphaviruses tested was reduced in C6/36.*w*AlbB cells compared to controls, across all MOI (Fig. 4). The magnitude of *Wolbachia*-mediated blocking of BFV (Fig. 4a–c) and SINV (Fig. 4d–f) replication increased with time. For BFV, this ranged from a reduction of 1–2 logs at 8–24 h post-infection (hpi) to more than 4 logs at 72–144 hpi in *w*AlbB-infected cells versus controls. BFV and SINV could be detected in culture supernatants for longer post-infection at high MOI rather than low MOI, although all viruses had disappeared from supernatants of *w*AlbB-infected cells by 144 hours into the experiment. At the MOI of 0.1, SINV could not be detected at 96 hpi; however, at the MOI of 10, replication was detected for a further 48 hours. RRV was largely undetectable at MOI of 0.1 and 1 (Fig. 4g–i), except for 8 hpi at MOI 1. However, at MOI of 10, infectious virus was detected until 32 hpi and thereafter only re-appeared at 72 hpi (Fig. 4i). There were no significant differences (general linear model *F*_(1, 6)_ = 2.33, *P* = 0.18) in the extent of *Wolbachia*-mediated blocking between flaviviruses and alphaviruses.

## Discussion

A large body of evidence has now accumulated documenting the ability of transinfected *Wolbachia* to block virus replication [50–52]. Although most reports have concerned the field-released *w*Mel strain, the ability of *w*AlbB to block virus replication is being increasingly explored. Our results show that yields of infectious virus from a range of flaviviruses were consistently reduced in *w*AlbB-infected C6/36 cells *versus Wolbachia*-free cells. Our data are consistent with previous reports of the ability of *w*AlbB to block ZIKV in other cell lines [53], although we observed much stronger blocking in the C6/36 system compared to this earlier report. It is also consistent with previous reports of DENV [40] and WNV_KUN_ [54] blocking in mosquitoes. Our data, using the C6/36 cell line background, confirm that the RNAi response is not an absolute requirement for *Wolbachia*-mediated blocking [55] since these cells are defective in this pathway [56]. Despite the reduction in virus replication observed due to *Wolbachia*, infectious DENV and WNV_KUN_ were produced and remained detectable in most treatments until the end of the experiment. By contrast, ZIKV levels rapidly fell below levels of detection for most MOI treatments and virus strains. The results suggest the blocking effect of *w*AlbB may be stronger for ZIKV than DENV, similar to observations from *Ae. aegypti* mosquitoes [40].

Significant blocking in *w*AlbB-infected cells was also observed for RRV, BFV and SINV compared to uninfected cells. This is similar to other studies utilizing alphaviruses, such as Semliki Forest virus [57]. In contrast to DENV and WNV_KUN_, infectious yields of alphaviruses in *w*AlbB-infected cells fell to undetectable levels much earlier in the experiment compared to control cells. The speed at which alphavirus stopped being produced in *Wolbachia*-infected cells was a function of inoculum size, with high MOI treatments producing detectable virus for much longer than low MOIs. For both BFV and SINV, we observed a ~ 24 h delay in the time taken for the MOI 10 infection to become undetectable in comparison to the MOI 1 infection. This delay due to higher initial inoculum was also observed with ZIKV, particularly the Asian genotype strains. Interestingly, the same pattern was not observed for DENV or WNV_KUN_. These data suggest that, for some viruses, the block hypothesized to occur early in infection, possibly at the virus translation stage [57–59], may be delayed if the initial virus population is large. A possible explanation is that a large starting population size allows the virus to partially overcome the initial challenge imposed by *Wolbachia* in these cells. However, subsequent cycles of infection may be hampered by low numbers of progeny viruses and the ability of *Wolbachia* to reduce the infectivity of these progeny [58, 60], ultimately causing extinction of the virus.

Our data show that differences in the ability of *w*AlbB to block viruses is related to individual virus species and strains rather than broader taxonomic groupings such as genera or families. For example, among the alphaviruses, RRV production was undetectable for most time points while BFV production was reduced at later time points (72–96 hpi). Within the flaviviruses, a similar pattern was observed for ZIKV, whereby the prototype strain MR766 was undetectable at most time points but Asian genotype strains persisted much longer, and, in some cases, infectious virus briefly rebounded from almost zero levels. These brief rebounds were also observed for the three alphaviruses, as well as WNV_KUN_, and were not always a function of high initial MOI. Subtle replication differences among virus species and strains [61] may result in varying abilities of arboviruses to persist and, potentially evade the blocking effect of *Wolbachia*.

## Conclusions

Our results have implications for using *w*AlbB to control arboviruses. As *w*MelPop appears unable to become established in wild mosquito populations [26] and *w*Mel may not survive at high temperatures in the field [62], alternative strains of *Wolbachia* need to be considered for biocontrol. Invasion of *w*AlbB-infected *Ae. aegypti* has been achieved for a small area in Malaysia [52] and has been associated with a reduction in the incidence of dengue in an endemic area [43]. Our study adds to the growing body of evidence that *w*AlbB is able to inhibit a wide range of mosquito-borne viruses and supports the case for a broader virus surveillance programmes in areas where the strain is being evaluated to determine whether it has an impact on diseases other than dengue.

## Data Availability

All data is presented within the paper and materials are available upon reasonable request.

## Abbreviations

BFV: Barmah Forest virus
CHIKV: chikungunya virus
CMC: carboxymethylcellulose
DENV: dengue virus
DTT: dithiothreitol
FBS: fetal bovine serum
FISH: fluorescent *in situ* hybridization
MOI: multiplicity of infection
PBS: phosphate-buffered saline
PFA: paraformaldehyde
RRV: Ross River virus
SINV: Sindbis virus
WNV_KUN_: West Nile virus (Kunjin strain)
ZIKV: Zika virus
